# Using simple technology to prompt multistep tasks in the home for people with dementia: An exploratory study comparing prompting formats

**DOI:** 10.1177/1471301215602417

**Published:** 2015-09-30

**Authors:** Hazel C Boyd, Nina M Evans, Roger D Orpwood, Nigel D Harris

**Affiliations:** Designability (Bath Institute of Medical Engineering), Bath, UK; Designability (Bath Institute of Medical Engineering), Bath, UK; Bath Institute of Medical Engineering, Bath, UK; Designability (Bath Institute of Medical Engineering), Bath, UK; University of Bath, UK

**Keywords:** prompting, sequencing, dementia, technology, assistive technology

## Abstract

**Objectives:**

To investigate the relative effectiveness of different prompts for people with dementia during multistep tasks in the home, to inform prompting technology design.

**Methods:**

Nine pairs of participants (one with dementia and a partner or relative) participated at home. The participants with mild to moderate dementia (5M/4F, aged 73–86 years) functioned at the Planned or Exploratory levels of the Pool Activity Level instrument. A touchscreen computer displayed different prompts during two set tasks: “card-and-envelope” and “CD player.” The trials were scored to establish the relative effectiveness of the prompts. Individual tasks were also explored.

**Results:**

Text and audio prompts were each more effective than video or picture prompts for a card-and-envelope task, but this was not seen in a CD player task. The differences may be related to the type of actions within the tasks; the card-and-envelope actions were easier to convey verbally; the CD player actions lent themselves to visual prompts.

**Conclusions:**

Designers of technology-based prompts for people with dementia should consider that the effectiveness of different prompts is likely to be task dependent. Familiar, unambiguous language can increase the success of tailored prompts. There are significant practical challenges associated with choosing and deconstructing everyday tasks at home.

## Introduction

People who have dementia typically experience deteriorating executive function, and particularly working memory, and therefore find it hard to complete multistep tasks (Wherton & Monk, 2010). It is generally accepted that enabling people to continue to perform activities that they were previously able to carry out is good for self-esteem. Indeed, providing a means of support for activities which involve several steps is considered to be important to the quality of life of people with dementia (Orpwood et al., 2007). Community-based occupational therapy is an effective way to improve quality of life and autonomy for people with dementia, as well as to support carers (Graff et al., 2007), but the resources available for providing such interventions are limited.

Technology could potentially provide support for multistep activities, as described by Dishman and Carillo (2007) and Hoey et al. (2012). A review of the efficacy of cognitive prostheses (Jamieson, Cullen, McGee-Lennon, Brewster, & Evans, 2014) concluded that it is possible for technology to improve confidence and support independence in people with memory impairments, but that such technology is not readily available in the same way as physical prostheses.

There is little comparative evidence on what types of prompts or formats should be used when providing this technological support.

Written labels and instructions are widely used in the home for people with (and without) dementia, but their effectiveness is reliant upon good reading ability and visual function. It should also be noted that labels and action prompts are not necessarily equivalent to each other and must be considered separately in terms of their intended function. Brennan, Giovannetti, Libon, Bettcher, and Duey (2009) found that written cue cards can actually make multistep tasks harder for people with dementia. The NeuroPage device successfully used text reminders sent to a pager (Wilson et al., 2009) as prompts for people with brain injury. However, this technology is not applicable to multistep tasks and, to be effective, the user must carry out the action shortly after receiving the prompt.

Prerecorded voice prompts have been used to provide information or instructions to a user in automated “smart homes” to initiate activities. This is a means of replacing the information that a carer might provide if they were present. Evans, Orpwood, Adlam, Chadd, and Self (2007) and Adlam, Evans, Gibbs, and Orpwood (2006) found that such voice prompts can positively influence the behavior of people with dementia in smart home environments. Audio information can also be useful for tasks that involve using the eyes and/or hands, and there is no need to move away from the task to take in a prompt. Lines and Hone (2006) found that natural voice recordings were preferred to synthetic speech outputs of interactive domestic alarm systems designed to support independent living in older people. Verbal audio prompts for people with cognitive impairments, including dementia, appear to be successful because of the strong link between executive function and verbal interaction (O’Neill & Gillespie, 2008). The General User Interface for Disorders of Execution (GUIDE) was developed for people with brain injury by O’Neill and Gillespie and can enable an individual to progress through a multistep task using audio voice prompts. The intention is to simulate naturalistic instructions and the system includes questions as well as prompts. The issue of when to provide prompts is an important aspect of the design of prompting technology. This has been addressed to varying degrees in the technology that has been designed to date; Wherton and Monk (2010) indicate that providing a prompt at the wrong time, for example, when an action has already been carried out, can be confusing. When audio prompts are used with older users, the volume and clarity of the recorded content also need to be considered. The primary limitation of audio prompts is that they are transient, i.e. when the audio information has finished, it is no longer available to the user.

Visual prompts in the form of pictures are widely accepted as a means of conveying ideas, or even complex instructions, for the general population. Perhaps the best example is the case of self-assembly furniture, where image-only diagrammatic instructions are common. There is not yet clear evidence that picture prompts are effective for people with dementia. Orpwood et al. (2007) carried out some exploratory comparisons of different formats in a simple sequence prompting device for making a cup of tea, and found that picture and video prompts seemed to encourage copying rather than understanding.

Video-based instructions are a logical progression from picture format prompts and are widely used in online guides through activities such as cooking and DIY. Video instructions to support people with dementia are used in the COACH system, developed by Mihailidis, Boger, Craig, and Hoey (2008) with a hand-washing task. This uses a video processor–based recognition system and voice recognition to identify individual steps with video content to provide the prompts. Ambient prompting technology*,* to enable people with dementia to carry out kitchen-based activities, is also being explored by Olivier, Xu, Monk, and Hoey (2009). The hardware includes accelerometers and Radio Frequency Identification (RFID) tags attached to kitchen items and video displays. These systems represent some of the most advanced technology in this field, but may not easily be applied flexibly to a range of activities in a home environment.

One key aspect of designing or assessing technology in this field is the difficulty of recreating “natural” conditions in which to try out types of prompting support. Controlled laboratory settings, although convenient for providing consistent test conditions, can also miss some of the relevant real-world factors that can impact upon a person’s ability to carry out a task. Indeed, Rusted and Sheppard (2002) showed that carrying out research in the familiar setting of people’s own homes, while less convenient and harder to control, can enable people with dementia to be more competent at carrying out tasks. The pressure of being watched while performing a difficult activity can also influence task ability, with users trying to “please” the researchers, or feeling anxious about being observed.

In addition to the issues described above, it is also clear that the level of prompting required for a given task will vary between individuals and for an individual over time, possibly even on a day-to-day basis as the dementia progresses. This may necessitate variable levels of prompting for the same person performing the same task.

The primary aim of this exploratory research study was to understand how to support a person with dementia through a multistep task, so that they could achieve the task independently. The long-term aim is to develop a simple, stand-alone prompting product that could be customized by a carer and enable a person with dementia to carry out multistep tasks independently at home.

The objectives were:

- To determine which formats of prompt were the most effective for delivering individual prompts for steps within the task, and reasons for their relative effectiveness
- To understand the effectiveness of combining different formats of prompts
- To investigate methods of moving from one prompt to the next as a person with dementia carries out tasks using technology prompts
- To explore the range of tasks that might be chosen by individuals in the home.

## Methods

Nine pairs of participants were recruited to the study via local interest groups and written, informed consent was obtained. Each pair comprised a person with dementia and a carer (typically a partner or son/daughter). The participants with dementia (5M/4F, 73–86 years) had a range of types of dementia and stages of progression. The diagnosis of dementia was reported by the carer, having been made by a clinician, e.g. a general practitioner. All were assessed at the start of their involvement in the study and were found to be functioning at the Planned or Exploratory levels in daily living and leisure activities, according to the Pool Activity Level (PAL) instrument (Pool, 2008). This scoring system was considered to be an appropriate measure to indicate their general level of functional ability and whether this had changed significantly during the course of the study. The PAL scores were determined by the occupational therapist (NE), based on the responses given by the carers to the PAL questionnaire at the start and end of the participants’ involvement in the study.

Potential participants contacted the researchers after reading an insert in the newsletter of a local dementia support organization (Alzheimer’s Support Wiltshire) and completing an expression of interest form about the study. The recruitment was carried out on a rolling basis.

Ethical approval for the work was granted by the University of Bath’s Research Ethics Approval Committee for Health (REACH). All participants with dementia were able to give written, informed consent at the start of the study, and consent was verbally confirmed at each visit.

Each visit took place at the home of the person with dementia, with the carer present. This enabled the carers to provide reassurance and allowed both participants to contribute to the study. All visits were carried out by an occupational therapist and a user interface engineer and each prompting test was video recorded to allow detailed observation to be carried out after each visit was concluded.

### Tasks and scoring

Two set tasks were used with each participant, plus one individual task that was personal to them. The first set task (“card-and-envelope”) was used with all nine participants, in order to compare how different types of prompts enabled them to perform the task. The task involved the following steps:

1. Take the card and sign it with your name
2. Put the card in the envelope
3. Put a stamp on the envelope.

This task was chosen as it was portable, nonmessy, suitable for both men and women, generally familiar, and could be broken down into a small number of steps. Some steps that might be carried out during this task, such as writing a greeting or addressing the envelope, were omitted from the set task in order to enable the researchers to see whether the participants were responding to the prompts or just working out how to do the task and guessing what was required.

Each time a test was carried out, a standard introduction script was used by the researchers to avoid giving any information that might affect their performance, e.g. “I’m going to set you a task to do, see how you get on; just follow the instructions on the screen.”

**Figure 1.**
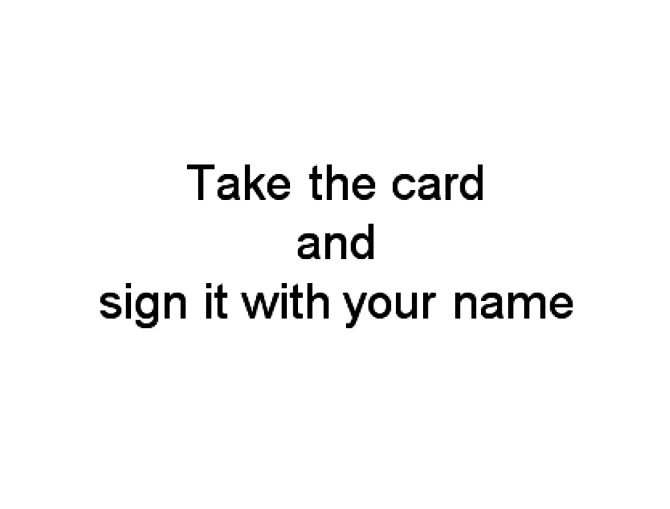
Example of a text prompt for the card and envelope task.

We evaluated four different types of prompts (given below). Each time a task was attempted, the participant was guided using only a single type of prompting format. For example, one trial might consist of the card and envelope task being carried out using a series of three text prompts. The four different formats of prompts were:

1. Text prompts (see example in Figure 1) displayed on a stand-alone touchscreen computer (an Asus Eee-top), positioned on a table in front of the participant with the materials for carrying out the task in front of the screen.
2. Recorded voice prompts containing the same information as in the corresponding text prompts, played by the same touchscreen computer near to the participant, but with the screen turned away.
3. Picture prompts displayed on the touchscreen. These comprised photos (from a head-camera view) of the exact materials provided to the participants with a pair of hands posed carrying out the relevant step.
4. Video prompts. These were short video clips, as per the picture format, of a pair of hands carrying out the action described in each step using the same materials available to the participants.

For this part of the work, one of the researchers would manually trigger the next prompt when they judged that the participant was ready.

The prompt formats were trialed in a randomized order to avoid any possible learning effect, although the researchers observed that the participants did not seem to remember the details of how to perform the activity from one visit to the next, even if they recognized the researchers and/or had some recollection of trying an activity on a previous occasion.

Where a participant was unable to carry out a step, progressive support was given by the researchers since each one required the previous one to be finished in order to carry it out; the ability of the participant to carry out each step using only the instruction (and not the researcher’s help) was accounted for in the scoring system described later. The range of support included reiterating the instruction using the same or different words, or pointing at the items, or occasionally physically demonstrating a step using the task materials provided.

**Figure 2.**
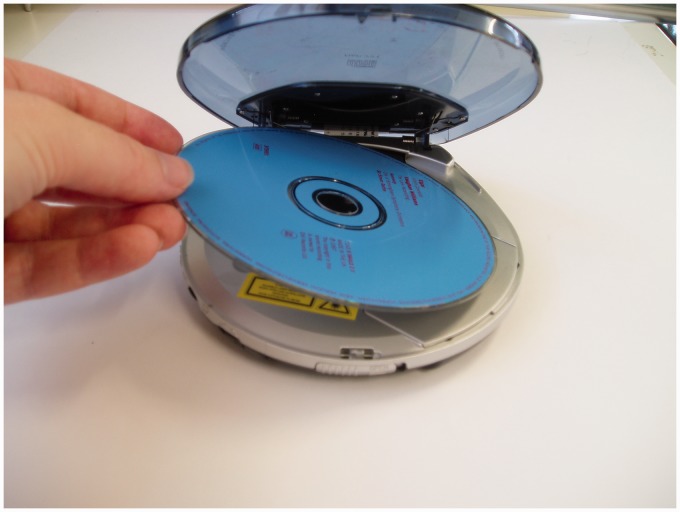
Example of a picture prompt for the CD player task.

A second set task (“CD player”) was developed and used with five of the participants, in order to investigate whether different tasks were influenced differently by the prompting formats. This task involved a portable CD player and trials were carried out as described previously (see Figure 2). The four task steps were:

1. Slide the switch on the front edge to open the lid
2. Put in a CD
3. Press the lid down gently to close it
4. Press the Play button to start the music.

These two set tasks were designed to enable direct comparison of the different types of prompts, and used later to observe the effects of enabling the participants to move through the prompts at their own pace, i.e. without the researchers choosing when to provide the next prompt.

A scoring system was developed to enable quantitative evaluation of the different formats and examine the effectiveness of each prompt to assess the success of the overall task. In contrast to tools such as the Perceive: Recall: Plan and Perform System (Nott, Chapparo, & Heard, 2009) which provide a detailed assessment of the cognitive deficit, the system used seven simple criteria for judging the participants’ behavior within each task. For each criterion the researchers judged whether it occurred always (score 2), sometimes (score 1), or never (score 0) for positive criteria, and with the scores reversed for negative criteria. These scores were then totaled for each trial to provide a measure of how successfully the participant had completed the task (see Table 1). “Diversionary behavior” is used here to describe distraction or face-saving behaviors, where the participant turns their attention away from the task by talking or asking about something unrelated to the task. Distraction by the detail of the task included behaviors such as commenting on the picture on the front of the greeting card or asking whether to write a greeting inside the card.

**Table 1.** Scoring table used to assess the set tasks (maximum possible score for completion was 14).

	Always (Score 2)	Sometimes (Score 1)	Never (Score 0)
Did they understand what each instruction was?			
Did they understand how to act on each instruction?			
Did they start each step using only the instruction?			
Did they complete each step using only the instruction?			

	Always (Score 0)	Sometimes (Score 1)	Never (Score 2)
Did they seek reassurance?			
Did they show diversionary behavior?			
Did they get distracted by the detail of the task?			

Following discussion with the person with dementia and their carer, one individual task was also chosen for each participant. The tasks were intended to be meaningful to the participants; some were chore-type activities and some were leisure related. This enabled observations to be made about some less well controlled but desirable task and inform the design of prompting technology for a wide range of activities. These tasks were approached in a more exploratory way. A user-led design approach was used to consider how prompts could best be used to support each activity drawing on knowledge gained from the first set task component of the study.

### Combining prompts

Alongside the comparisons between the different prompting formats, we also carried out some exploratory work to investigate the effect of combining some types of prompts, to understand how we could enable the participants to move to the next prompt at an appropriate time.

## Results

At the start of the study, all participants had been functioning at Planned or Exploratory levels according to the PAL. Seven of the nine participants had a PAL score recorded 2.5–8 months later on completion of the study, of which 3 showed a change in scores moving slightly or substantially toward or into the adjacent activity level, indicating a possible progression of their dementia.

### Comparing prompt formats during the two set tasks

The video footage from each attempt at the card and envelope task was observed and the scoring system applied to each attempt. For consistency, all of the scoring was carried out by one researcher (HB). The other researcher (NE) and an independent occupational therapist were also asked to independently score subsets of the results. The prompting format scores were ranked in the same order by all three observers. The individual scores are shown in Table 2.

**Table 2.** Individual scores for all participants for four prompting formats for the card-and-envelope task (maximum possible score for completion was 14).

Participant	A	B	D	I	K	L	M	O	Q	Mean score
Text	10	12	12	11	14	9	14	11	14	11.9
Picture	4	6	7	9	7	9	8	6	6	6.9
Audio	–	–	9	13	14	10	14	12	13	12.1
Video	4	5	5	10	9	5	9	5	14	7.3

Each participant tried each format only once, because of the practical and resource challenges in carrying out batteries of tests in the participants’ home environment. Each visit was carefully planned to minimize participant fatigue as this could have affected the results of the testing. The order of the formats was varied between participants, so that any learning effects, or potential progression of their dementia, would not bias the results. A total of 67 visits were made to participants during the course of the study. The visits included combinations of single-format tests for one or two standard tasks, plus exploration of individually chosen tasks, as well as some study of combinations of prompts and demonstrations of examples of how future designs of such prompting technology might look. Typically there were a total of three short activities (trials or explorations) per visit.

Two audio prompt results were missing: one was not video recorded and the other could not be delivered due to a hearing impairment. A univariate analysis of variance showed there were significant differences between the effectiveness of the different prompt formats for the envelope task (*p *< 0.001). Post hoc analysis (Tukey) showed that the text and audio formats were each significantly better than the picture or video formats (*p *< .01).

The relative effectiveness of the text and audio messages was considered to be because the audio information was easy to take in, being similar to the spoken prompting that a carer might naturally provide, and because the text information was unambiguous and was particularly suited for the first step of the task (“Take the card and sign it with your name”). This was harder to convey using some of the other types of prompt. The success of the text prompts was a surprise to some of the carers, as they described how their loved ones “Did not read any more,” as the activity of reading had become too difficult or not satisfying (possibly because of the concentration required to understand news content or fictional plots). However, it was clear that every participant with dementia could still read functionally and act on the information provided to them if it was delivered clearly and concisely.

Picture prompts were seen to be less effective than text or audio prompts, even though the prompts were photographs of the actual items and layout as the items provided to carry out the task. It was noted that some participants took some time interpreting the photo prompts to work out what was in the image, or even trying to recreate the image by carefully positioning their hands and the objects in a static pose. This could have been caused in part by the researchers inadvertently using the phrase, “Just copy what’s on the screen,” or, “Just copy the picture” to support participants, but this did not seem to be the cause in every case. This caused sufficient distraction for some participants not to be able to complete the task easily; consequently pictures that were in any way ambiguous were considered not to be useful prompts.

The video prompts were not always effective. The primary reasons for the lack of effectiveness seemed to be: (i) as with the pictures, some interpretation was needed, particularly for step 1 of the task, which required the participant to understand that they should sign their name, rather than write a greeting or address a person, (ii) the video clips were not long, but the participants had to remember the content of the video clip in order to carry out the step, (iii) there may have been a tendency for video content to be viewed passively and not acted upon, perhaps because of the participants’ usual experience of recorded video content on television or films. The researchers had to replay the clips on some occasions, indicating the weakness of this format (and indeed the audio format) because of its transient nature. Replaying the video clips was not considered to bias the scores for the video format, because the scores related to the use of the video prompt on the first occasion; subsequent plays were only used to enable the participant to move to the next step.

The second set task (CD player) was chosen to determine whether the type of task, and the individual steps within the task, had an influence on the relative effectiveness of the different formats of prompts. The two tasks comprised different types of actions and this was reflected in the results. Table 3 shows the scores across five participants for the four prompting formats.

**Table 3.** Mean scores for four prompting formats for the CD player task for five participants (maximum possible score for completion was 14).

Participant	K	L	M	O	Q	Mean score across all participants
Text	11	10	11	9	9	10
Picture	10	9	11	8	13	10.2
Audio	11	10	11	10	13	11
Video	9	8	10	11	13	10.2

The scores for the CD player task were similar across the different formats and the text and audio prompts were not shown to be any more effective than picture and video prompts.

The first step of the CD player task (“Slide the switch on the front edge to open the lid”) was the hardest to perform. This was because the lid of the CD player was sprung and would only open when the switch was used correctly and the lid was not inadvertently being held down. This was a complex step to explain using only words, as was the use of the Play button; the participants generally understood to look for the (labeled) Play button, but text and audio prompts were not the best way to convey, in only a few words, where to find the Play button in the array of similar-looking buttons.

The lid-closing step was easier to convey using words, so text and audio prompts were helpful for this step. However, the step, “Put in a CD” elicited a wide range of responses from the participants, probably based on their prior experience of using CDs.

### Exploring the individually chosen tasks

Individual tasks were chosen following discussion between the researchers and each pair of participants. One of the main challenges was that some of the participants with dementia were no longer very active at home, so it was not easy to find a suitably meaningful activity. The researchers worked with the pairs of participants to deconstruct the task into steps and developed sets of prompts in suitable formats to support each person with dementia through their own task. This was done using a user-led design approach, where the designs could be adapted based on their effectiveness. A wide range of approaches was used, based on the individual activities and circumstances. A summary of these prompts and findings is given in Table 4.

**Table 4.** Details of and findings from individually chosen tasks.

Individual task (task origin)	Initial formats and delivery methods used	Key findings	Considerations for technology development
Operating a series of switches to prepare a Wii games console to play tennis (task created by researchers, since participant was very inactive)	Touchscreen computer gave picture and audio prompts about how to operate each switch	This participant tried to operate a switch on the prompting screen, having mistaken this for the button on his own television	Prompting devices can be confused with task-related appliances
Emptying the dishwasher (task usually done daily by participant with support from spouse)	Touchscreen gave audio messages and onscreen text messages	Meaningful wording can be hard to get right; the prompt “Dry the tops of the cups” was initially effective, but in a subsequent session, the participant commented, “But those are the bottoms of the cups!”	Use of the right language is essential
Getting a preprepared supper from the fridge at tea time (meal usually left in fridge daily so the participant could eat before daughter arrived, but food not usually eaten without prompting)	For one step, a stand-alone movement sensor was triggered when the participant entered her kitchen, triggering an audio message saying, “Your tea is in the fridge.”	Activities that include moving between rooms can be more complex to prompt, even using just audio prompts, as the information must reach more than one location. For some people, well-timed audio messages can be effective	Location and timing of prompts is important
Putting music on one’s own CD player built into a hi-fi system (task usually done alone by participant by trial and error with varying success)	This participant was provided with a text and image-based “Manual” for his CD player, with one prompt in large print per page	The images were removed, as they were perceived as “irrelevant”. This participant could work his own CD player by trial and error and did not follow the text prompts in order; some people do not tend to use a stepwise, methodical approach to tasks	Personality and usual approaches to tasks can influence the success of prompting
Switching on the radio (a new task agreed by request to relieve anxiety, since switching on the radio was desirable but not done by participant when alone)	This participant was sometimes anxious while waiting for his partner to arrive home, so audio prompts and a labeled one-button radio were provided so that he could switch on the radio himself	Some tasks could be used to pass time or alleviate anxiety. Some tasks need to happen at a set time; an “alarm” function to trigger task support could be beneficial	Automatically timed suggestion of tasks by a prompting device could be beneficial
Making a cup of tea for two people (task usually done together daily by participant and husband, or by participant with varying support from husband)	Text and audio prompts provided on touchscreen. This participant was a very inexperienced technology user, so the visual content was designed to look like an open recipe book, with a front cover at the start entitled, “Tea for Two”	This task comprised many steps, even using relatively coarse steps such as “Get the milk out of the fridge.” The participant was able to carry out all of the individual steps, but was very unsure about using technology. The recipe book visual format was more engaging for this participant than any suggestion of using “The screen” or “the device”	Touchscreens can be made more engaging by using appropriate metaphors
Changing the TV channel (TV watched daily in daughter’s absence, but changing channel more successfully could give improved autonomy of viewed content)	The initial approach was to use a stand-up flip-over photo holder, with text and picture prompts. The subsequent approach was to use a single A4 sheet standing up in a menu holder, with all of the steps visible	The steps included pointing the TV remote the right way round at the TV with a direct line of sight from the TV remote to the TV. Turning over the physical pages of the photo album required dexterity and disrupted the flow of the task. The wording was changed a few times, to aid understanding. During the course of this study, the participant learnt some of the steps, namely the pointing the TV remote correctly, and subsequently required fewer prompts	Some required task steps are not obvious without very close observation. Use of the right language is essential Learning is possible and support can be reduced, as well as increased, over time
Loading a weekly medication dosette box with support from a carer (task usually done together, with wife providing prompting to participant to carry out some supervised steps)	Both participants shared this task. Audio and text prompts were used to support the participant with dementia to open the relevant compartments, and his partner passed him the required pills	This participant was not confident about the idea of the prompting, but was very capable at following the instructions methodically, having had been a teacher in a technical field. The use of precise wording to convey ideas was important in this task: the prompt, “Open all of the top windows” was an effective way of conveying the idea of opening all of the boxes that contained morning tablets	Personality and usual approaches to tasks can influence the success of prompting. Use of the right language is essential
Locking the front door from the inside at night (task attempted each night with some prompting from wife, and achieved with varying degrees of success)	This participant had a new, unfamiliar, double-glazed front door. A set of audio prompts was recorded using a single three-prompt recording with appropriate gaps in between them on a “Talking Tile” located by the front door	On subsequent visits, this participant sometimes did not need to use the prompts at all in order to lock the front door. The phrase “Turn the key to the left” was effective, where “anticlockwise” was not	Learning is possible and support can be reduced, as well as increased, over time. Some tasks are suited to a series of fixed-time prompts. Use of the right language is essential

### Exploring self-forwarding for set and individual tasks

The need for end users of prompting technology to be able to move from one prompt to the next was briefly explored for the set and individual tasks. The initial set task single-prompt tests were all carried out with a researcher discreetly using a mouse or keyboard away from the touchscreen to deliver each prompt. However, any practical device would clearly need some way of triggering the next prompt without another person being present. In this exploratory study, we considered that a simple approach, rather than say a computer vision approach, was desirable. To test this out, a simple “Next Step” button was designed to appear on the touchscreen, allowing users to move to the next step in their own time, once they had finished the previous step. A delay was built in so that the button only appeared after a few seconds. This delay enabled the user to see and take in the prompt for each step without initially being distracted by the idea of moving to the next prompt.

Different options were used for the wording of the button. These included, “Touch here for next step” and “Next step.” For the individual task making tea for two, this was changed to “Next page” or “Touch here for next page,” to be consistent with the recipe book metaphor. The button-like appearance of the on-screen button was considered to be useful, although for some participants, it was important also to include, “Touch here” in order to mitigate any unfamiliarity with using touchscreens. Other participants could manage well with less information if they could remember that the screen was a touchscreen. This feature highlighted the common design compromise required when designing for people with dementia: the tension between adding extra words for clarity (“Touch here for next step”) and the need for brevity (“Next step”). The button was kept in the same location (bottom right) throughout to provide consistency for the users. The bottom-right position also reflected the usual last place on a page that the eye is drawn when reading Western text.

One valuable finding from the use of the self-forwarding feature was that in at least three cases, the participants were able to carry out some steps of a task without the prompts (i.e., they got ahead of the prompting sequence), but this did not impair their ability to carry out the task. Indeed, at least one participant said that they knew they had already completed one of the on-screen steps without a prompt and pressed the button for the next prompt, and they then noted that they had also completed this further prompted step, before arriving at a step that corresponded to their current place in the task.

### Looking ahead: Using combinations of prompts and self-forwarding together

Combinations of more than one prompt format and the self-forwarding feature were integrated into revised series of prompts for the set tasks and for some individual tasks and some preliminary observations were made about their use. The combinations of prompt types had to be carefully tailored to different tasks and for different users. Combining two or more formats of prompt was seen to be particularly effective, particularly where one static visual (e.g., picture or text) and one word-based (e.g., audio or text) prompt were used together. One particularly strong combination was text and recorded voice audio prompts. The primary strength in combining visual and audio information is that spoken words are strongly connected to executive function and on-screen text information can be referred back to and mitigates the transient nature of the spoken words. There is clearly also scope for including picture-based prompts in combination with word-based prompts such as text or audio, because so many appliances and consumer goods in the home rely on successfully identifying different buttons and switches.

As expected, these advanced prompts were found to be more effective than the single prompts alone and the participants were sometimes able to achieve a whole task in one go without external help.

## Discussion

### Comparing prompt formats during the two set tasks

The comparison of four different ways of delivering prompts during the first set task showed that text or audio (voice recorded) prompts were a clear and effective means of enabling a person with dementia to carry out the actions required to complete simple tasks in the home. Picture prompts required too much interpretation and therefore proved distracting if used alone. Video prompts were short-lived and again needed some interpretation. The same types of prompts used for a CD player task, which relied heavily on finding and operating buttons and switches, gave different results. Some of the actions required in the CD player task were more readily described using pictures and harder to convey using text.

The card and envelope task contained actions and objects that the participants were familiar with. This familiarity would be expected of a task that someone with dementia might try to retain (rather than learning a completely new activity) if using a complete prompting system. However, the participants found the use of a portable CD player, or similar technology, was less consistently familiar between the participants, and none had used that exact model of CD player before. The card task contained steps that could easily be described unambiguously using words, given the familiar nature and limited options afforded by the objects for that task. The most challenging step to describe in words was “take the card and sign it with your name,” but this action was familiar enough to all of the participants that no further detail was required. However, this was hard to describe using a picture or video alone, eliciting comments such as “What shall I write?” and “What does that say?” Some participants wrote extra content, or a greeting, during this step when no verbal instructions were used. The CD player task involved two steps that were hard to convey as ideas to novice users using words: lifting the lid by sliding a sprung switch while not holding down the sprung lid accidently; and putting in the CD and pressing it down gently, which required careful location of the center hole of the CD onto the spindle. Closing the lid was simple to convey, but pressing the Play button to start the music was easier to perform accurately when a picture was used to show which of the similar, silver buttons was the play button (even though the play button was labeled with the word “Play” as well as the standard play symbol).

Two participants provided an interesting contrast relating to their familiarity with using CD players. While the particular model of portable CD player used in this study was unlikely to have been seen by any of the participants, one person had worked using music CDs in a former job, while another had never used a CD player and found the task more difficult. Prior experience may reflect on their ability to carry out the task, although it must be highlighted that the intended function of this type of prompting technology is, in the first instance, to enable people to maintain familiar activities.

### Exploring the individually chosen tasks

The study of individually chosen tasks demonstrated that identifying and deconstructing appropriate activities are not trivial. The different types of tasks, and indeed the steps within them, lent themselves to different types of prompting format.

The tasks included chore-type activities, as well as leisure activities; the primary concern was to explore tasks that the participants felt would be meaningful to maintain and the researchers did not aim to steer the choice of activity into any particular category. The main considerations for developing technology were the precise use of meaningful language within the prompts and ability to break down the tasks into suitable steps.

### Exploring self-forwarding for set and individual tasks

We found that providing a simple means of enabling the users to move to the next prompt in their own time worked well. Providing a forwarding button after a short delay of a few seconds mitigated any possibility of distracting the user from the content of the current step. The simplicity of this approach was considered to be justified by the observation of participants “getting ahead” of the prompts and yet still successfully carrying out task steps in order and completing activities. This finding was very encouraging, bearing in mind the aim of providing simple prompting technology for familiar tasks.

### Looking ahead: Using combinations of prompts and self-forwarding together

The early exploration of integrated features provided promising insight into the potential for an independently usable prompting system in the home. The self-forwarding approach trialed here was considered to be a suitable basis for the design of this aspect of a technology prompter. Using combinations of prompting formats was also observed to be effective, although this aspect will require more human intervention to tailor the specific combinations of format required in each situation.

### Limitations of the study

The artificiality of the testing, including the use of tasks that have different levels of familiarity to different people, is an inevitable limitation of the study and one that was mitigated to some extent by the inclusion of self-chosen, meaningful tasks. The researchers were keen to focus on the ultimate aim of the work, which was to support people to maintain familiar, meaningful tasks, rather than to be able to perform new, unfamiliar tasks.

The home setting enabled the researchers to experience the living environment of the participants, enabling a rich understanding of the factors needed to design appropriate technology for the home. The participants can also be more at ease at in their own home environment than in a laboratory setting. However, this setting can provide additional distractions for the participants and is resource-intensive for the researchers.

The small number of participants allowed a detailed understanding of the individuals and of some of the design requirements for a home-prompting system. A larger, qualitative study of a fully developed prompting system would be valuable as a follow-on study, since there are several aspects of a market-ready prompting product which need further exploration. These include the practical implications of asking a carer to identify and deconstruct suitable tasks, and the usability of any interfaces needed when setting up the prompts.

The resources and the potential fatigue of the participants meant that only one trial could be carried out per prompt format and no repeated data were gathered. However, the study was carefully managed to minimize the effects of learning or the progression of the participants’ dementia.

The recruitment method did result in some heterogeneity of the participants, which was considered to be acceptable within the scope of this initial proof-of-concept study. However, the effect of this was mitigated somewhat, because in the researchers’ experience, the most likely users of assistive technology will be those who have a carer or relative who is interested in supporting and encouraging its use. As such the self-selecting group seen here could be representative of those end users.

The results were dependent on the exact way that the task steps were conveyed to the participants; other studies might use subtly different content that could influence the results. However, when combined with the researchers’ observations of the individually chosen tasks, the type of task action being described and the prompting format did appear to be the primary determinant of how easily each participant was able to complete that step.

## Conclusions

Studying two set tasks in order to compare different formats of prompts led the researchers to the following conclusions. Recorded voice audio prompts reinforced by text prompts are powerful in combination and can be well understood. Each of these two prompt formats alone also has potential for prompting in this context. The language used within the prompts must be explored carefully for each individual in order to make it meaningful. However, the type of task and the type of action being carried out do sometimes require the use of images. Image prompts alone should be used with caution, as without further clues their interpretation can be disruptive to the flow of a task. Actions that can be unambiguously conveyed using words, and which involved familiar objects or controls, were well suited to word-based prompting. Actions that involved choosing between visually similar objects or controls, or which required specific, fine motor actions could benefit from the addition of a picture prompt.

The long-term aim of using such a technology-based prompting device should be to retain tasks that are already familiar to the user, thereby eliminating some of the challenges associated with understanding what each step involves and focusing instead on clear, timely reminders of previously understood steps.

Exploring individually chosen tasks using a user-led design approach highlighted the complexity of applying the findings from the set tasks to real-life situations. Choosing meaningful tasks, and breaking them down in a way that a carer would usually provide prompting support, is not trivial. The physical or environmental context of the task can also affect the flow of the task, and the personality of the person carrying out the task can influence how methodically the prompts are followed.

This study revealed some of the design and practical challenges surrounding the development of prompting technology for people with dementia carrying out tasks at home. The findings from this study should be of interest to designers of prompting systems for people with dementia, or any technology where prompting is required as part of another goal.
